# Gastric venous congestion and bleeding in association with total pancreatectomy

**DOI:** 10.1002/jhbp.523

**Published:** 2017-12-19

**Authors:** Akimasa Nakao, Suguru Yamada, Tsutomu Fujii, Haruyoshi Tanaka, Kenji Oshima, Yukiko Oshima, Kiyotsugu Iede, Hironobu Kobayashi, Yasunori Kimura, Yasuhiro Kodera

**Affiliations:** ^1^ Department of Surgery Nagoya Central Hospital 3‐7‐7 Taiko Nakamura‐ku, Nagoya Aichi 453‐0801 Japan; ^2^ Department of Gastroenterological Surgery (Surgery II) Nagoya University Graduate School of Medicine Nagoya Aichi Japan

**Keywords:** Gastric bleeding, Gastric venous congestion, Pancreatic cancer, Portal vein resection, Total pancreatectomy

## Abstract

**Background:**

Gastric venous congestion and bleeding in association with total pancreatectomy (TP) were evaluated.

**Methods:**

Thirty‐eight patients of TP were retrospectively analyzed. TP was classified as TP with distal gastrectomy (TPDG), pylorus‐preserving TP (PPTP), subtotal stomach‐preserving TP (SSPTP), and TP with segmental duodenectomy (TPSD).

**Results:**

Portal vein or superior mesenteric vein resection and reconstruction was performed in 24 patients (62.2%). Gastric bleeding occurred immediately after tumor resection in one of eight patients who underwent SSPTP, and urgent anastomosis between the right gastroepiploic and left ovarian vein stopped the bleeding. Another case of gastric bleeding was observed a few hours after TP in one of nine patients who underwent PPTP, and hemostasis was achieved after conservative therapy. Gastric bleeding was not observed in 16 patients who underwent TPDG and five who underwent TPSD. Some patients underwent preservation of gastric drainage veins (left gastric vein, right gastric vein, or right gastroepiploic vein). Neither patient with bleeding underwent preservation of a gastric drainage vein.

**Conclusions:**

To preserve the subtotal or whole stomach when performing TP, one of the gastric drainage veins should undergo preservation or reconstruction, and anastomosis between the right gastroepiploic vein and left ovarian vein may be beneficial.

## Introduction

Total pancreatectomy (TP) is sometimes indicated for treatment of chronic pancreatitis or tumors of the whole pancreas. TP is usually combined with distal gastrectomy to reduce gastric venous congestion and bleeding secondary to difficulties with preservation of the gastric drainage veins. The indications for gastrectomy or preservation of the whole or subtotal stomach combined with TP and gastric venous reconstruction are not clear 1, 2, 3, 4, 5, 6, 7. Since 1993, we have encountered two patients with gastric bleeding among 37 patients treated with TP. Therefore, a retrospective study was conducted to clarify the risk factors for gastric venous congestion and bleeding.

## Methods

**Figure 1:**
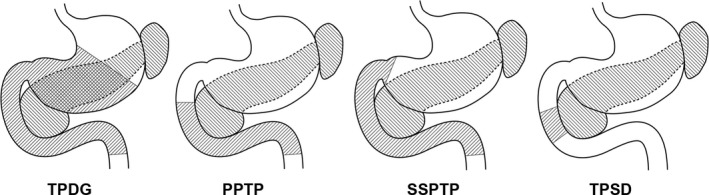
Types of total pancreatectomy according to the type of gastrectomy (1993–2015). *PPTP* pylorus‐preserving total pancreatectomy, *SSPTP* subtotal stomach‐preserving total pancreatectomy, *TPDG* total pancreatectomy with distal gastrectomy, *TPSD* total pancreatectomy with segmental duodenectomy

A total of 38 patients underwent TP at the Department of Surgery II, Nagoya University and Department of Surgery, Nagoya Central Hospital from 1993 to 2015. These 38 patients comprised 26 with pancreatic adenocarcinoma, seven with intraductal papillary mucinous neoplasms, two with metastasis of renal cell carcinoma, two with neuroendocrine tumors, and one with chronic pancreatitis. There were 20 men and 18 women with a mean age of 60.9 (range, 39–78) years. The TP procedure was classified according to whether resection or preservation of the stomach was performed: TP with distal gastrectomy (TPDG), pylorus‐preserving TP (PPTP), subtotal stomach‐preserving TP (SSPTP), and TP with segmental duodenectomy (TPSD) 8, 9 (Fig. 1). Vascular resection and reconstruction of the portal vein (PV) or superior mesenteric vein (SMV) was performed in 24 patients. No arterial resection and reconstruction was performed in these patients. When a long time was required to resect and reconstruct the PV or SMV, the PV catheter‐bypass procedure 10, 11 was performed using an antithrombogenic catheter to prevent portal congestion during clamping of the PV or SMV under the non‐touch isolation technique using the mesenteric approach 12, 13. Preservation of the gastric drainage veins was classified as follows: preservation of no drainage veins (Type 0), preservation of the left gastric vein (LGV) (Type LG), preservation of the right gastric vein (RGV) (Type RG), and preservation of the right gastroepiploic vein (RGEV) (Type RGE). All patients were checked for gastric venous congestion and bleeding during surgery and postoperatively until discharge from the hospital.

This clinical research was approved by the institutional reviewer board (H29‐039).

## Results

No mortality occurred among the 38 patients who underwent TP. PV or SMV resection was performed in 24 (63.2%) of the 38 patients, and a catheter‐bypass procedure was used in 13 of these 24 patients. All 38 patients were classified according to their operative procedures. The performance of PV or SMV resection and the types of gastric drainage vein preservation are listed in Table 1.

**Table 1:** Operative procedures of TP, PV or SMV resection, types of gastric drainage vein preservation, and gastric bleeding

Operative procedures	Number of patients	PV or SMV resection	Types of gastric drainage vein preservation					
			0	LG	RG	RG+LG	RG+RGE	RGE
TPDG	16	14	15	1	0	0	0	0
PPTP	9	1	1 (1*)	5	2	0	1	0
SSPTP	8	8	2 (1*)	6	0	0	0	0
TPSD	5	1	0	1	2	1	0	1
Total	38	24	18 (2*)	13	4	1	1	1

Data are presented as numbers of patients. The number in parentheses with an asterisk indicates the number of gastric bleeds.

*PPTP* pylorus‐preserving total pancreatectomy, *PV* portal vein, *SMV* superior mesenteric vein, *SSPTP* subtotal stomach‐preserving total pancreatectomy, *TP* total pancreatectomy, *TPDG* total pancreatectomy with distal gastrectomy, *TPSD* total pancreatectomy with segmental duodenectomy, *Type 0* no preservation of gastric drainage veins, *Type LG* preservation of the left gastric vein, *Type RG* preservation of the right gastric vein, *Type RGE* preservation of the right gastroepiploic vein

Gastric bleeding from the nasogastric tube was observed in two (5.3%) of the 38 patients who underwent TP. The first patient with bleeding was a 56‐year‐old man with pancreatic head cancer, and pylorus‐preserving pancreaticoduodenectomy was performed. Development of a carcinoma in the pancreatic body and tail from the pancreatic head was rapidly diagnosed by pathological analysis of frozen sections of the cut surface of the pancreas. Therefore, PPTP was finally performed. The PV and SMV were preserved in this patient; however, the gastric drainage veins, including the LGV, RGV, and RGEV, could not be preserved. We observed slight gastric venous congestion without bleeding from the nasogastric tube during the operation. However, postoperative bleeding from the nasogastric tube was observed 2 to 3 h after closure of the abdominal wound. Gastrointestinal fibroscopy was immediately performed, and venous congestion and bleeding from the esophageal and gastric mucous membranes were observed. However, hemostasis was successfully achieved in this patient by conservative therapy.

**Figure 2:**
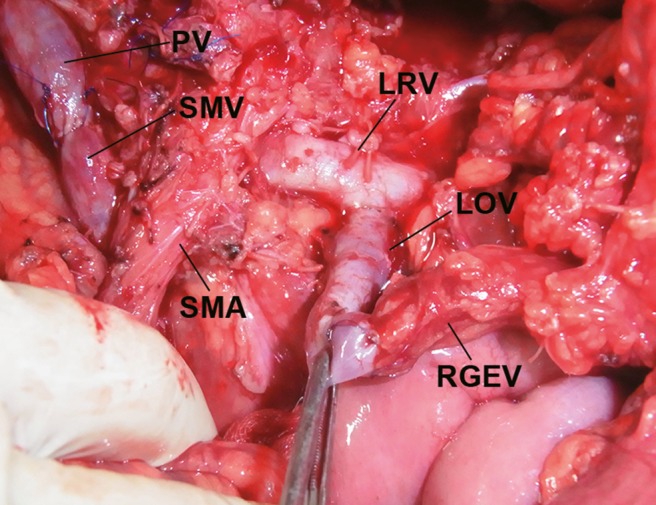
Anastomosis between the RGEV and LOV in subtotal stomach‐preserving total pancreatectomy with PV and SMV resection. *LOV* left ovarian vein,*LRV* left renal vein, *PV* portal vein, *RGEV* right gastroepiploic vein, *SMA* superior mesenteric artery, *SMV* superior mesenteric vein

The second patient with bleeding was a 64‐year‐old woman with a pancreatic neuroendocrine tumor. The PV, SMV, and splenic vein were completely obstructed by tumor invasion, and collateral veins developed. SSPTP with PV and SMV resection and reconstruction was performed in this patient. Severe gastric venous congestion and bleeding from the nasogastric tube were observed immediately after resection of the tumor. A large dilated and congested RGEV was observed. The left ovarian vein, which flows into the left renal vein, was exposed and divided. The peripheral side of the left ovarian vein was ligated, and end‐to‐end anastomosis between the RGEV and proximal side of the left ovarian vein was conducted (Fig. 2). The gastric venous congestion disappeared immediately, and the bleeding from the nasogastric tube was stopped. The subtotal stomach was preserved; however, none of the gastric drainage veins were preserved (Type 0). One patient who underwent SSPTP (Type 0 procedure) who did not develop gastric bleeding had no gastric venous congestion during the surgery. The only difference in peri‐operative parameters between the former two patients with bleeding and the latter patient with no bleeding was macroscopic gastric venous congestion during the surgery.

**Figure 3:**
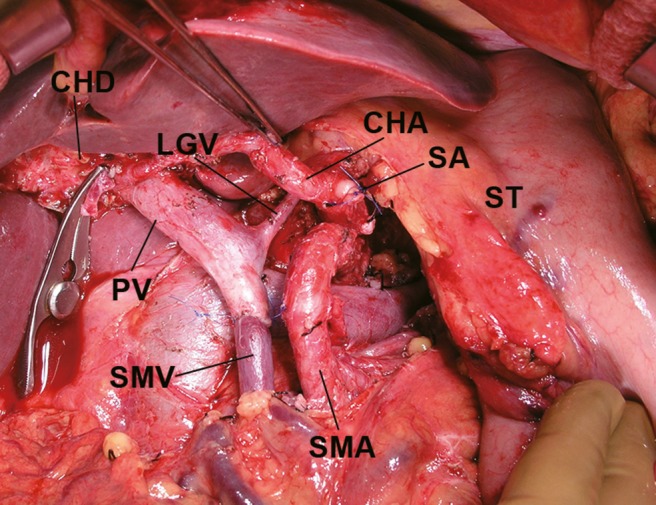
Preservation of the LGV in subtotal stomach‐preserving total pancreatectomy with PV and SMV resection. *CHA* common hepatic artery, *CHD* common hepatic duct, *LGV* left gastric vein, *PV* portal vein, *SA* splenic artery, *SMA* superior mesenteric artery, *SMV* superior mesenteric vein, *ST* stomach

**Figure 4:**
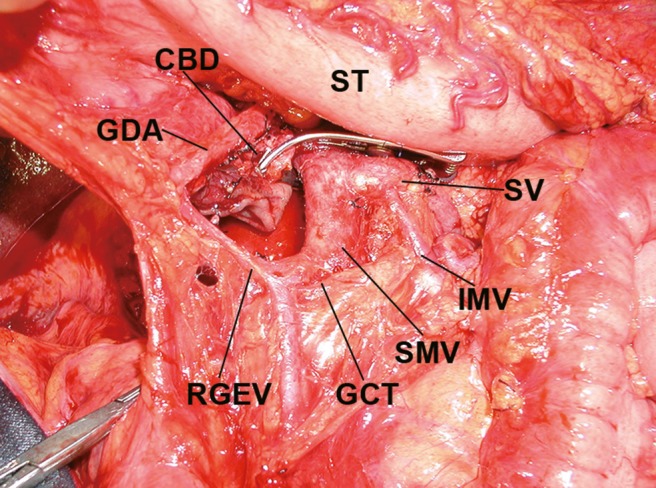
Preservation of the RGEV in total pancreatectomy with segmental duodenectomy. *CBD* common bile duct, *GCT* gastrocolic trunk, *GDA* gastroduodenal artery, *IMV* inferior mesenteric vein, *RGEV* right gastroepiploic vein, *SMV* superior mesenteric vein, *ST* stomach, *SV* splenic vein

Sixteen patients who underwent TPDG had no gastric bleeding despite the fact that PV or SMV resection was performed in 14 of these patients and 15 underwent Type 0 procedures. Special attention was recently given to preservation of one of the gastric drainage veins, such as the LGV (Fig. 3) or RGEV (Fig. 4), when we performed PPTP, SSPTP, and TPSD.

## Discussion

The development of catheter bypass of the PV using an antithrombogenic catheter 10, 11 removed the time limitation associated with PV obstruction during surgery. We have been performing isolated pancreatectomy combined with PV or SMV resection using a mesenteric approach 6, 12, 13. When we performed TP in the present study, we routinely carried out distal gastrectomy at the outset. Many studies of TP did not pay close attention to gastric venous congestion. However, we have been trying to preserve the subtotal or whole stomach as much as possible since 1993. During the past 22 years, we have experienced two patients with gastric bleeding due to severe gastric venous congestion after TP. Distal gastrectomy is an easy way to reduce gastric venous congestion and bleeding. However, a wide range of gastrectomy procedures combined with TP will worsen patients’ nutritional conditions. When TP is planned for treatment of pancreatic tumors or chronic pancreatitis, close attention must be given to the gastric drainage veins. PV or SMV resection was performed in 24 of 38 patients who underwent TP in the present study; however, the LGV was preserved in 13 of these patients. Sometimes TP is necessary after pancreatoduodenectomy because of carcinoma invasion from the head to the body and tail of the pancreas. In the present study, gastric drainage veins such as the LGV, RGV, and RGEV were already resected before additional resection of the pancreatic body and tail, along with the spleen. Therefore, distal gastrectomy was necessary in these patients without the gastric drainage veins. Distal gastrectomy is one procedure with which to reduce gastric venous congestion of the remnant stomach in association with TP. In one patient of SSPTP with Type 0 who did not develop gastric bleeding might be considered the left phrenic vein or well developed submucosal venous plexus in esophago‐gastric junction. In the one patient who underwent SSPTP (Type 0 procedure) who did not develop gastric bleeding, the remnant veins were considered to be the left phrenic vein or the well‐developed submucosal venous plexus within the esophagogastric junction. These drainage routes were likely sufficient to drain the remnant stomach. When we perform distal pancreatectomy for pancreatic body cancer, carcinoma invasion into the pancreatic head is sometimes observed 14. In this situation, the LGV, RGV, and RGEV are candidates for possible preservation, and TPSD is one of the good indications for preservation of the whole stomach 9, 15 (Fig. 4). We encountered only one patient who underwent reconstruction of a gastric drainage vein (Fig. 2). Few reports have described reconstruction of the gastric drainage veins 16, 17; however, it is a difficult procedure. The anatomy of the gastric drainage veins should be determined by preoperative computed tomography angiography 18, 19, 20 and intraoperative inspection. The importance of preservation of the LGV 21, 22, 23, 24 or RGEV 15 has been emphasized in previous reports. Preoperative or intraoperative planning for preservation of the gastric drainage veins is important when TP is performed.

A limitation of the present study is its small sample and retrospective design. We only performed exploratory analyses of the feasibility and short‐term outcomes of this procedure with respect to gastric venous congestion and bleeding by categorizing the patterns of gastric and venous resection. This topic should be investigated in a multi‐institution study with a larger sample.

In conclusion, distal gastrectomy may be a safe method with which to prevent gastric venous congestion and bleeding when combined with TP. To preserve the subtotal or whole stomach, one of the gastric drainage veins should undergo preservation or reconstruction. Anastomosis between the RGEV and left ovarian vein is a new operative procedure to reduce gastric venous congestion.

## Conflict of interest

None declared.
